# ADAM8 in squamous cell carcinoma of the head and neck: a retrospective study

**DOI:** 10.1186/1471-2407-12-76

**Published:** 2012-02-27

**Authors:** Valerie Zielinski, Markus Brunner, Gregor Heiduschka, Sven Schneider, Rudolf Seemann, Boban Erovic, Dietmar Thurnher

**Affiliations:** 1 Department of Otorhinolaryngology, Medical University of Vienna, Vienna, Austria; 2 Department of Otorhinolaryngology and Maxillofacial Surgery, Medical University of Vienna, Vienna, Austria; 3 Department of Otorhinolaryngology, Head and Neck Surgery, Medical University of Vienna, Waehringer Guertel 18-20, A-1090 Vienna, Austria

**Keywords:** ADAM8, Squamous cell carcinoma, Head and neck

## Abstract

**Background:**

A disintegrin and metalloproteinase (ADAMs) have been associated with multiple malignancies. ADAMs are involved in cell fusion, cell migration, membrane protein shedding and proteolysis. ADAM8 has been found to be overexpressed in squamous cell carcinomas of the lung. A new study showed that ADAM8 is significantly overexpressed in metastasis of squamous cell carcinomas of the head and neck (HNSCC).

**Methods:**

We determined ADAM8 levels in the serum of 79 HNSCC patients at the time of diagnosis, in 35 patients 3 months after treatment and in 10 patients 1 year after therapy and compared the results to the sera of 31 healthy volunteers. We also constructed tissue microarrays to detect ADAM8 immunohistochemically in 100 patients. The results were correlated with the survival data of the patients to determine the diagnostic and prognostic value.

**Results:**

The data demonstrated that patients with high ADAM8 expression in the tumor have worse survival rates. We found that high ADAM8 serum levels correlated with high ADAM8 expression in tumor samples. Soluble ADAM8 levels did not show any prognostic or diagnostic properties.

**Conclusion:**

In summary ADAM8 expression is a prognostic factor for survival of patients with head and neck squamous cell carcinoma.

## Background

Worldwide more than half a million patients are diagnosed with HNSCC [1]. The global mortality rate is 6/100.000 [2]. The incidence of head and neck cancer in Austria, according to the Austrian Federal Institute for Statistics (Statistik Austria) is approximately 1000 per year. With a ratio between 2:1 and 4:1 males are affected more than women. Currently, the most commonly used prognostic and predictive factors are the TNM staging system (AJCC, 6th Edition Cancer Staging Atlas) and the presence of HPV/p16 [3]. Additional reliable predictive or prognostic tests would be very helpful to determine the best type of treatment for our patients.

"A disintegrin and metalloproteinase"(ADAMs) are a family of proteins, which are similar to the reprolysin family of snake venomases. These reprolysin families share the metalloproteinase domain with matrix metalloproteinases (MMPs) [4].

ADAMs are known to play a role in cell-cell and cell-matrix interactions through the disintegrin domain [5]. ADAMs are cell surface and extracellular multidomain proteins involved in cell-cell signaling, cell adhesion and cell migration, cell fusion, membrane protein shedding and proteolysis [4]. They therefore are thought to promote tumor growth [6,7]. Matrix metalloproteinases have been identified to play a role in the infiltration of tumors into the surrounding tissue by having the ability of destroying extracellular matrix including the basement membrane [8].

Overexpression of ADAM8 has been shown in renal cell carcinomas, in pancreatic cancer [9], in prostate cancer [6] and in primary brain tumors [10]. In this current study we wanted to identify the prognostic value of ADAM8 serum and tumor levels in HNSCC.

Ishikawa et al. showed that in squamous cell carcinomas of the lung ADAM8 was significantly overexpressed compared to a healthy control group [11]. They also showed that transfection of ADAM8 into tumor cells elevated the invasiveness [2]. The aim of the study was to determine the relevance of the protein in HNSCC. Therefore we investigated ADAM8 serum levels and tumor expression in tumor patients and correlated the results to patients' clinical data.

## Methods

### Patients

In our study 148 patients with squamous cell carcinoma of the head and neck were included prospectively, all diagnosed at the Department of Otolaryngology of the Medical University of Vienna. The inclusion criterion was the presence of a previously untreated, histologically proven squamous cell carcinoma of the oropharynx, hypopharynx, oral cavity or larynx. Exclusion criteria were infectious diseases, immunosuppression and malignancies other than squamous cell carcinoma of the upper respiratory tract.

All patients underwent a physical examination with special attention to the head and neck region, a panendoscopy, a complete blood count, a biochemical analysis of liver and kidney function and electrolytes, an electrocardiogram, chest x-rays, an abdominal ultrasound, a computed tomography scan of the head and neck and the medical history were obtained.

The tumor samples were taken during diagnostic panendoscopy. Blood samples were taken at the time of diagnosis, 3 months and 12 months after the end of therapy, respectively. In addition, serum of 31 healthy volunteers who were selected to approximate the age range in patient samples served as control.

Tumor samples from 100 patients were available for immunohistochemical analysis. Tumor and blood samples were taken from 31 patients and used to compare immunohistochemistry to Elisa results. The median observation period was 32 months (range 25-40 months). Of the 148 patients included, 66 were primarily operated, 79 received primary radiotherapy and 3 refused treatment.

The patients were primarily Caucasian and male. There were 31 female patients and 117 male patients. Of the 79 patients whose serums were analyzed 11 were women and 68 were men. The carcinomas which were included were distributed as follows: 30 Hypopharynx carcinomas, 24 carcinomas of the oral cavity, 34 carcinomas of the tongue, 25 carcinomas of the Larynx, 35 Oropharynx carcinomas including 18 carcinomas of the tonsils. The patients primarily had advanced tumor stages. 50 patients had a T1/T2 carcinoma, 98 had a T3/T4.

Radiotherapy was performed using external beam irradiation with a final dose of 72 Gy in fractions of 1.8 Gy. Chemotherapy consisted of Cisplatin 100 mg/m^2^/day on weeks 1 and 3.

All samples were obtained after informed consent and collected using protocols approved by the Institutional Review Board.

### Tissue microarray

Before analysis, hematoxylin-eosin-stained sections from each tumor sample were reevaluated and the suitability of inclusion in the study was determined. Sections of 2 to 3 μm were used for the analysis. The suitability of the tissue was evaluated using a number of inclusion criteria such as the size (1-2 mm in depth and at least 5 mm in length and width), as well as other features, such as appropriate fixation, absence of significant electrosurgical device lesions, signs of acidic decalcifying agents, and the presence of usable tissue in each block. Each hematoxylin-eosin-stained slide was reevaluated and mapped to identify the specific areas for tissue acquisition to build the tissue microarrays using a manual tissue arrayer (MTA-1; Beecher Instruments, Sun Prairie, WI). Core diameter was 0.6 mm. 3 cores were used per patient. We constructed tumor tissue microarrays from 100 paraffin embedded tumor samples.

### Immunohistochemistry

Before analyzing the samples the optimal dilution ratio and best choice of retrieval buffer was determined. To determine the expression of ADAM8 we stained the tissue microarrays with a commercially available antibody specific for ADAM8.

Paraffin embedding was removed from the microarray slides and after rehydration they were subjected to antigen retrieval in a microwave oven (600 W) employing Tris-EDTA buffer followed by 3 wash cycles with TBS buffer for 5 min. Unspecific binding was avoided by adding 5% TBS/BSA for 1 h at room temperature. Then the primary antibody (ADAM8, 1:100, mouse IgM) (MBL, USA) was applied and incubated overnight at 4° in a wet chamber. A sample without primary antibody was used as negative control. The next day the secondary biotinylated antibody (1:200, Multilink, Dako, DK) in 1% TBS/BSA was applied and incubated for 1 h at room temperature. After another washing cycle alkaline phosphatase conjugated Streptavidin-AP/TBS/BSA (1:250, Dako, DK) was applied for 1 h at room temperature. Visualization was performed by fast red (Sigma, Missouri, USA) and counterstained by haemalaun. Samples were analyzed using an Olympus BH-2 microscope.

Afterwards three independent investigators (M.B., B.E., S.S.) analyzed the staining intensity by assigning each to one of 3 levels. 0 is no staining, 1 is moderate staining, and 2 is strong staining. Observer bias was avoided by repeating the evaluation of protein expression at two different time points and without knowledge of patients' clinical data. Mean IS was calculated from the three samples per patient.

### ELISA

Serum ADAM8 levels were determined in 79 head and neck cancer patients before treatment, in 35 patients after treatment and in 10 patients 1 year after therapy and in 31 controls by using a commercially available enzyme test kit (R&D System Inc., USA).

The manufacturer's instructions were strictly followed. As a control one well was used without antibody. In short: after coating a 96-well micro plate with a monoclonal antibody specific for ADAM8 and incubated over night, the sera diluted 2:1 with the reagent diluent was added and allowed to incubate for 2 h at room temperature. After washing off the unbound substances the detection antibody was added, incubated for 2 h. After another washing step Streptavidin-HRP was added and incubated for 20 min. At the end the substrate solution was added to the wells. The color-reaction was stopped after 20 min by adding 50 μl of 2 NH^2^SO4.

The color reaction in the different wells was measured by a microplate reader set to 450 nm with a wavelength correction of 540 nm.

### Statistical analysis

The overall significance level was set to be 0,05. The remaining significance level was distributed to 5 hypothesis [12].

To calculate the prognostic survival of the immunohistochemistry results the "Logrank-Test"was used and then shown in a Kaplan-Meier curve. To compare the ADAM8 serum levels of the 4 different groups a Kruskal-Willis rank sum test was used.

To test for trend of ADAM8 serum levels with rising immunohistologic grade of tumor samples a Jonckheere-Terpstra test was carried out. The difference of ADAM8 serum concentration between early and advanced patients was determined by a Wilcoxon rank sum test with continuity correction. This test was also used to evaluate if there was a difference in the serum level of ADAM8 between the healthy control group and patients with HNSCC. To determine the prognostic value of ADAM8 serum levels a Wilcoxon rank sum test was used.

## Results

### Clinical data

In total 148 patients were included in the study of which 27 were women and 121 were men. The median age was 58 years (range 21-88 years) for the patients and 57 years (range 39-81 years) for the control group.

At time of diagnosis blood samples were taken from 79 patients, after 3 months 35 patients were available for a second serum sampling and after 1 year there were 10 patients for a third serum sampling. The remaining patients could not be analyzed for the following reasons: 12 had died, 6 were lost for follow up and the remaining were not able to come to our department in time for blood sampling.

**Figure 1:**
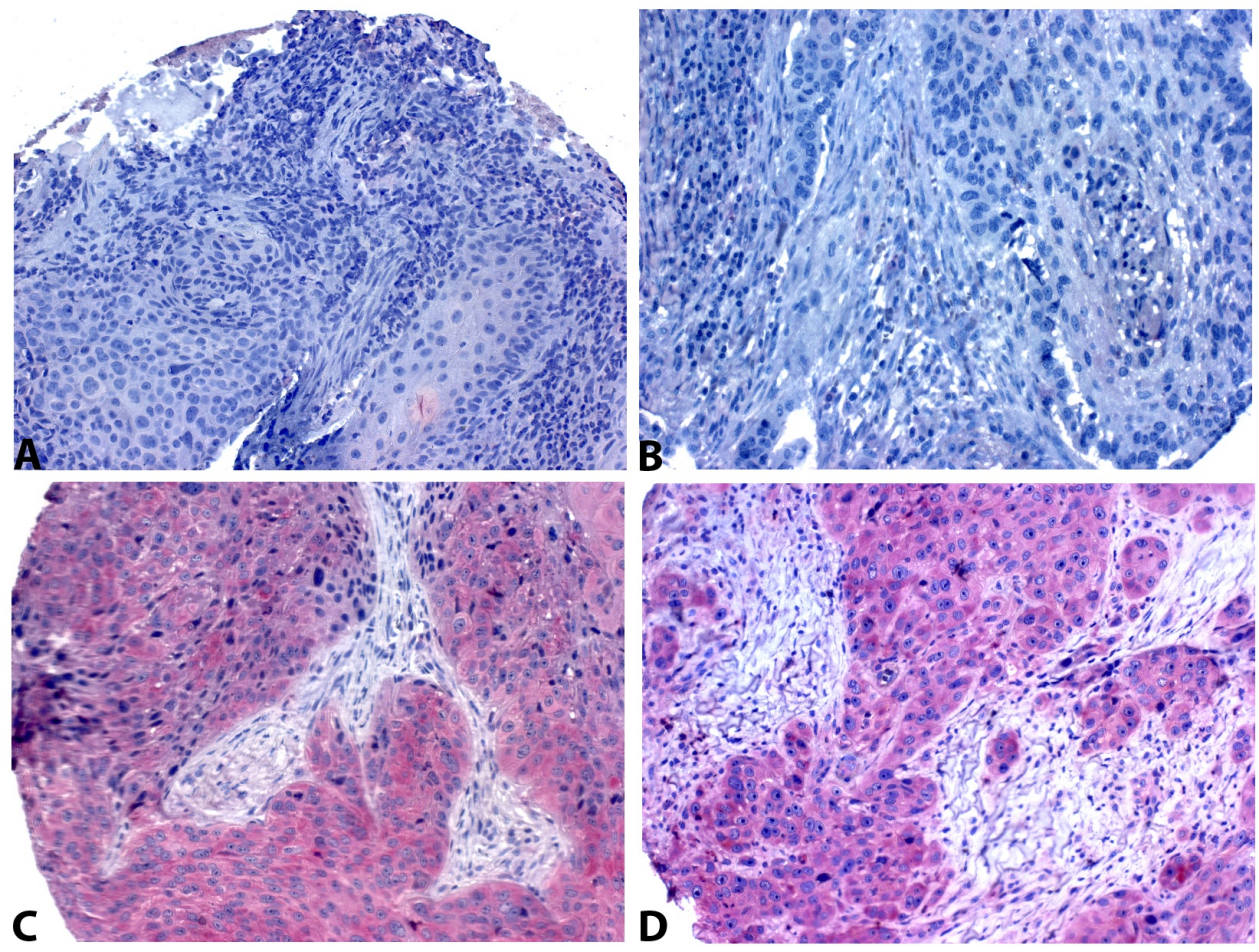
**Pictures of immunohistochemical staining of ADAM8, 200 × magnification**. A and B: ADAM8 negative, C and D: ADAM8 positive.

**Figure 2:**
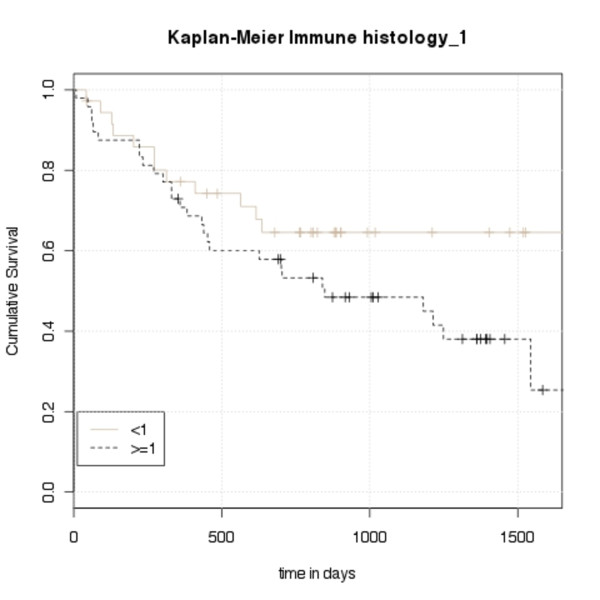
**Kaplan- Meier Curve of immunohistochemistry results divided in two groups (staining < 1 and staining ≥ 1)**. 0 represents no staining, 1 represents moderate and 2 represents strong staining. Mean IS was calculated from the three samples per patient.

**Figure 3:**
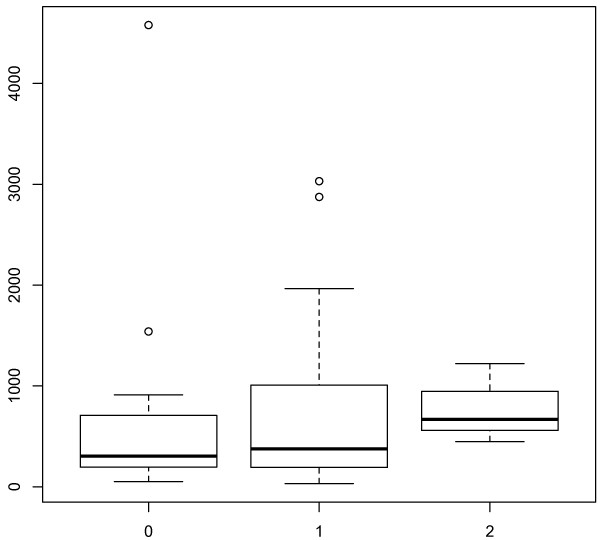
**Correlation of immunohistochemistry results and ELISA results**. X-Axis: Immunohistochemistry staining. Y-Axis: Elisa results at time of diagnosis.

The statistical analysis showed that expression levels of ADAM8 in immunohistochemistry corresponded with survival (*p*-value = 0.011, JT = 4380). Examples of the immunohistochemistry staining can be found in Figure 1. Patients with tumors overexpressing ADAM8 have significantly worse survival (see Figure 2). ADAM8 serum levels in the ELISA correlated well with immunohistochemistry expression levels, (*p*-value = 0.019). The correlation of the results of immunohistochemistry and ELISA is shown in Figure 3. We, however, did not find a correlation between soluble ADAM8 levels and patients' survival (*p*-value = 0.128).

We could not demonstrate a significant difference between ADAM8 serum levels of patients with HNSCC at the time of diagnosis compared to the healthy controls.

(*p*-value = 0.325). The comparison of soluble ADAM8 in early (T1/2, N0) and advanced (T3/4, N+) tumor stages did also not reveal a significant difference.

**Figure 4:**
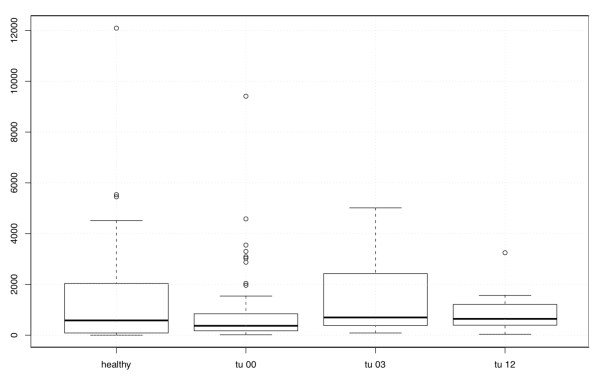
**Boxplots of the serum levels of ADAM8 in all 4 groups**. Healthy represents the ADAM8 expression in the healthy control group N = 31, tu00 represents the ADAM8 expression in the serum of tumor patients at the time of diagnosis, N = 79, tu03 represents the ADAM8 expression in the serum of tumor patients 3 months after treatment N = 35, and tu12 represents the ADAM8 expression in the serum of tumor patients 12 months after treatment N = 10.

There was a difference of ADAM8 serum levels at different stages of disease when comparing pre-therapeutic patients, patients 3 months after therapy and patients 12 months after therapy (*p*-value = 0.010). The healthy control group and patients 3 months after therapy had higher ADAM8 serum levels than patients at the time of diagnosis and 12 months after therapy (see Figure 4).

Immunohistochemistry results were divided as follows: 51 patients had no staining, 45 had moderate staining and 4 had strong staining.

## Discussion

In the prospective study we analyzed the utility of ADAM8 as a diagnostic and prognostic tool in patients with head and neck squamous cell carcinomas.

Squamous cell carcinomas of the head and neck have a high morbidity and mortality rate mainly because of their high invasive and metastatic potential and high recurrence rates. The prognosis and the quality of life of patients with SCC of the head and neck have improved, but not as much as in other entities. The often late diagnosis of these cancers is one of the main problems. A diagnostic blood test to facilitate early tumor detection would therefore be of great importance.

Metalloproteinases have been shown to play a role in the infiltration of tumors into surrounding tissue. Since HNSCCs have an especially high tendency for infiltration these proteinases have been a focus of investigation. HNSCC spread to cervical lymph nodes and result in distant metastasis, high rate of recurrence and high risk of second malignancies [8]. Stokes and colleagues were the first to associate ADAM8 with HNSCC [13]. They found that ADAM8 was expressed significantly higher in metastatic tumors as well as identifying several proteinases of the ADAM-family (ADAM9, ADAM17, ADAM28, ADAMTS1, ADAMTS8 and ADAMTS15) -including ADAM8- which are HNSCC associated [13]. To date the clinical value of ADAM8 is still unknown.

Our study was the first to compare serum and tumor levels of ADAM8 in the same patients with HNSCC. We found that elevated serum levels of ADAM8 corresponded with high expression of ADAM8 in tumor samples (*p*-value = 0.011, Jonckheere Terpstra = 4380).

The survival analysis of our study clearly demonstrated that a high ADAM8 expression in the tissue sample correlates with earlier death. As seen in the Kaplan Meier curve (Figure 2) strong ADAM8 staining in the tumor is associated with worse survival rates. Unfortunately, in contrast to tumor expression, serum levels of ADAM8 did not predict survival.

However, in this preliminary study the number of patients was too small for a subgroup analysis of tumor location and treatment type. To find a difference between these subtypes a much larger group of patients and probably a multi-center study would be necessary.

## Conclusions

In summary tumor ADAM8 expression is a prognostic marker for survival in contrast to serum levels. Patients with high ADAM8 expression had a worse outcome than patients with lower ADAM8 expression. These preliminary results are promising but the clinical significance of this finding has to be determined in larger studies.

## Abbreviations

ADAM8: A disintegrin and metalloproteinase domain 8; AJCC: American joint committee on cancer; HNC: Head and neck cancer; HPV: Human papillomavirus; N: Lymph nodes; M: Metastasis; SCC: Squamous cell carcinoma; T: Tumor; TNM: Tumor lymph node and metastasis staging; MMP: Matrix metalloproteinase.

## Competing interests

The authors declare that they have no competing interests.

## Authors' contributions

VZ data acquisition, wrote the manuscript and performed the ELISA. MB, SS and BE evaluated the immunohistochemistry. RS carried out the statistical analysis. GH helped perform the ELISA. DT designed and coordinated the study and helped draft the manuscript. All authors read and approved the final manuscript.

## Pre-publication history

The pre-publication history for this paper can be accessed here:

http://www.biomedcentral.com/1471-2407/12/76/prepub
